# Validation of Next Generation Sequencing Technologies in Comparison to Current Diagnostic Gold Standards for *BRAF*, *EGFR* and *KRAS* Mutational Analysis

**DOI:** 10.1371/journal.pone.0069604

**Published:** 2013-07-26

**Authors:** Clare M. McCourt, Darragh G. McArt, Ken Mills, Mark A. Catherwood, Perry Maxwell, David J. Waugh, Peter Hamilton, Joe M. O'Sullivan, Manuel Salto-Tellez

**Affiliations:** 1 Molecular Pathology Programme, Centre for Cancer Research and Cell Biology, Queen's University Belfast, Belfast, United Kingdom; 2 Haematology Programme, Centre for Cancer Research and Cell Biology, Queen's University Belfast, Belfast, United Kingdom; 3 Prostate Cancer Programme, Centre for Cancer Research and Cell Biology, Queen's University Belfast, Belfast, United Kingdom; 4 Belfast Trust, Belfast City Hospital, Belfast, United Kingdom; 5 Cancer Science Institute Singapore, National University Health System & National University, Singapore, Singapore; Children's National Medical Center, Washington, United States of America

## Abstract

Next Generation Sequencing (NGS) has the potential of becoming an important tool in clinical diagnosis and therapeutic decision-making in oncology owing to its enhanced sensitivity in DNA mutation detection, fast-turnaround of samples in comparison to current gold standard methods and the potential to sequence a large number of cancer-driving genes at the one time. We aim to test the diagnostic accuracy of current NGS technology in the analysis of mutations that represent current standard-of-care, and its reliability to generate concomitant information on other key genes in human oncogenesis. Thirteen clinical samples (8 lung adenocarcinomas, 3 colon carcinomas and 2 malignant melanomas) already genotyped for *EGFR, KRAS* and *BRAF* mutations by current standard-of-care methods (Sanger Sequencing and q-PCR), were analysed for detection of mutations in the same three genes using two NGS platforms and an additional 43 genes with one of these platforms. The results were analysed using closed platform-specific proprietary bioinformatics software as well as open third party applications. Our results indicate that the existing format of the NGS technology performed well in detecting the clinically relevant mutations stated above but may not be reliable for a broader unsupervised analysis of the wider genome in its current design. Our study represents a diagnostically lead validation of the major strengths and weaknesses of this technology before consideration for diagnostic use.

## Introduction

Molecular cancer diagnostics in clinical practice is constantly and rapidly evolving. With the need to identify standard-of-care mutations in companion diagnostics to predict therapeutic response, cancer treatment has been revolutionised.

Since the 1970s, the Sanger method [1] is the gold standard for mutation analysis in cancer diagnostics; however its low-throughput and relative low sensitivity, long turnaround time and overall cost [2] have called for new paradigms. Next Generation Sequencing (NGS) can massively parallel sequence millions of DNA segments and, in principle, offers benefits relating to possible lower costs, increased workflow speed and enhanced sensitivity in mutation detection [3]. As whole-genome-sequencing may be unaffordable for routine diagnostics, the targeted sequencing of exon coding regions or a subset of ‘genes of interest’ offered by NGS is an attractive proposition a priori [4]. When compared with single-gene analysis, the use of NGS in diagnostics would allow the analysis of more than one therapeutic avenue as well as the generation of other valuable information for research purposes. To be a plausible option, a) NGS technologies must be as efficient as the current detection methods in the diagnosis of those single genes that currently represent standard-of-care; and b) the extra information generated must be of sufficient quality to consider alternative therapies or be accepted for downstream research endeavours. Such validations should include, at least, the V600E mutation in the *BRAF* gene, indicating which malignant melanoma patients respond effectively to vemurafinib treatment [5], [6], mutations of the *EGFR* gene to predict which lung adenocarcinomas respond to tyrosine kinase inhibitor (TKI) treatment, primarily those identified in amino acid (aa)719 exon 18, exon 19 deletions, aa768 exon 20 and aa858 exon 21 [7], [8], and *KRAS* mutations in exon 2 (codon 12 and 13) to predict lack of response to targeted monoclonal antibodies in colorectal cancer [9]. To test the presumed advantage of NGS versus single-gene approaches, the bench validation must be accurately executed and major challenges in bioinformatic analysis met. Indeed, the clinical utility of NGS has been described in other disease settings. For example, NGS is as reliable as Sanger sequencing in the detection of a range of mutations associated with hereditary cardiomyopathy. The authors concluded that targeted NGS of a disease-specific subset of genes is equal to the quality of Sanger sequencing and it can therefore be reliably implemented as a stand-alone diagnostic test [10].

Here, we test the validity of the current technical designs for the NGS analysis of *BRAF, EGFR* and *KRAS* (and of more than 40 other key oncogenes), exploring all the bench and bioinformatics analytical variables to confirm the possible application of these technologies for routine cancer diagnostics.

## Materials and Methods

(See S1 for choice of clinical materials, DNA extraction protocols, sequencing analysis by Sanger and q-PCR platforms, general sequencing workflow for NGS analysis and ethical framework of the study). Thirteen aliquots of tumour DNA extracted from formalin-fixed-paraffin-embedded (FFPE) malignant melanoma, lung adenocarcinoma and colon carcinoma, and genotyped by Sanger/q-PCR sequencing for BRAF, EGFR and KRAS status, respectively, were obtained from the Northern Ireland Biobank following ethical approval (NIB12-0049). The data set has been deposited in NCBI SRA http://www.ncbi.nlm.nih.gov/sra (SRP023265).

### IonTorrent sequencing

PGM sequencing was performed according to IonTorrent protocols using 10 ng DNA (Table S1), IonAmpliSeq™Cancer Panel primer pool and IonAmpliSeq™Library Kit 2.0 Beta (Life Technologies) for whole-exon-sequencing of *BRAF, EGFR* and *KRAS* and targeted “hot-spot” regions in 43 other cancer-related genes (http://tools.invitrogen.com/content/sfs/brochures/IonAmpliSeq_CancerPanel_Flyer_CO32201_06042012.pdf). Template preparation was performed on the Ion OneTouch™ system for 100 bp libraries (Life Technologies). QCs were performed using the IonSphere™Quality Control Kit (according to the protocol) ensuring that 10-30% of template positive Ion spheres were targeted in the emPCR reaction. Prior to loading onto 314 chips, sequencing primer and polymerase were added to the final enriched Ion spheres.

### 454 GS Junior sequencing

GS Junior Titanium Fusion primers for BRAF (exon 15) and KRAS (exon 2) were designed incorporating 8 Roche multiplex identifier (MID) barcodes. BRAF and KRAS libraries were prepared adhering to the Roche Amplicon Library Preparation manual. For EGFR, libraries were prepared using the EGFR18-21MastR kit (Multiplicom), according to the accompanying protocol. Clonal amplification onto DNA capture beads was performed manually adhering to the emPCR Amplification manual-Lib A (Roche). After DNA library bead enrichment, adaptor-specific sequencing primers were annealed and libraries were sequenced according to the Sequencing Manual on the GS Junior (Roche).

### Bioinformatics analysis

#### IonTorrent ‘closed’ bioinformatics

was performed using IonTorrent Version (V) 2.0.1 (ID.1-ID.10 and ID.12) and re-analysed by V2.2 upgrade (all samples). The HG19 reference was used for alignment. For 314 chip sequencing a threshold of ≥200,000 final quality library reads was applied.

#### GS Junior ‘closed’ bioinformatics

was performed using Roche 454 Amplicon Variant Analyzer (AVA) V2.7 for BRAF and KRAS. HG19 *BRAF* and *KRAS* regions were used as the alignment reference, respectively. Multiplicom provided scripts for analyzing their EGFR18-21MastR Kit sequencing results. A threshold of ≥50,000 final quality library reads was applied for all. Variants obtaining a frequency of detection ≥5% were considered in the analysis.

#### ‘Open’ CLC Genomics Workbench

V5.5 was employed to comparatively analyse data generated by IonTorrent software (PGM) and AVA (GS Junior). HG19 was downloaded within CLC, incorporating tracks from the COSMIC database. Alignment was carried out by 2 methods: a loose alignment setting to identify large base changes, for example deletions, and a stringent alignment setting for quality based variant detection (QBVD) of single nucleotide variants (SNVs), both capped at 5% mutation frequency. Three QBVD thresholds were applied: (i) the lowest coverage and (ii) the second lowest coverage required to detect standard-of-care variants. A third arbitrary threshold (iii) at ∼2-fold higher coverage than (ii) was also employed. Coverage equated to (i) = 71 (aa719 exon 18; EGFR), (ii) = 259 (aa858 exon 21; EGFR) and (iii) = 500, meaning any SNVs with coverage equal to or above these thresholds were included in CLC analysis and compared. For IonTorrent, four sets of data were retrieved: V2.0.1, CLC_V2.0.1 (ID.1-ID.10 and ID.12), V2.2 and CLC_V2.2 (all samples), and for GS Junior, 2 sets: AVA and CLC_AVA. Ultimately, a variant ‘passed’ if detected in 2/4 of the resultant analyses enabling a consensus list of SNVs and standard-of-care genes across different software tools and versions. Concordance (rho.c value) analysis was performed evaluating the frequency of variants detected against the reference between the following groups: CLC_IonTorrentV2.2 vs IonTorrentV2.2; Ion TorrentV2.2 vs AVA; AVA vs CLC_AVA.

## Results

### EGFR analysis

#### Lung adenocarcinoma (ID.3-ID.10)

Deletion in exon 19 affecting aa745_750 was detected in 25% of patients (ID.7 and ID.10) and was concordant across Sanger, q-PCR (Cobas), IonTorrent (V2.2) and Roche GS Junior platforms (mean coverage depth >2500, Table 1). A variant in exon 20 G/A aa803 was detected in ID.10 by IonTorrent but disregarded as re-analysis by CLC and subsequent GS Junior sequencing did not confirm the base call hence not meeting our variant ‘passed’ criteria outlined in Materials and Methods. Lowering the stringency of the bioinformatic analysis allowed the detection of standard-of-care TKI-sensitising mutations [7], [8] that were not detected by single gene analysis and thus, are likely to represent false-positive results. For example, in ID.9, a SNV was detected in exon 18 G/T aa719 by IonTorrent and, as a result of applying the lowest threshold, this SNV was also detected by CLC re-analysis at coverage of 89. By employing more stringent thresholds (QBVDii = 259; QBVDiii = 500), this standard-of-care mutation was not detected. Variants that ‘passed’ the 2/4 analysis criteria but not identified by the gold standard Sanger/q-PCR methods were in ID.4, ID.5 and ID.9 by IonTorrent (and CLC re-analysis) at the sensitizing mutation EGFR exon 20 G/T aa768 [8]. This finding was discordant with GS Junior and Sanger/q-PCR. Sequencing coverage averaged at 477 (for all 3 QVBD thresholds). Of note, when applying the highest threshold (QVBDiii = 500) only, this standard-of-care mutation would only have been called in ID.4. Variant ‘passed’ in 4/4 NGS analysis was the important activating mutation in ID.9 at exon 21 T/G aa858 (also identified by Sanger/q-PCR). Importantly, by applying the highest QBVDiii threshold of 500, this key SNV would only have been called in 1/4 analysis of ID.9. Variants ‘passed’ in 4/4 NGS analysis were the two standard-of-care mutations in exon 18 G/T aa719 and exon 20 G/T aa768 in ID.8, concordant with Sanger/q-PCR (Table 1). Even when applying the most stringent threshold (QBVDiii = 500), both therapeutic mutational targets were called. In addition, two silent germline polymorphisms [10], [11] were detected in 4/4 NGS analyses: a SNV at exon 21 C/T aa836 in ID.9 and a SNV at exon 20 G/A aa787 in all lung adenocarcinoma samples. Frequencies of EGFR SNVs were concordant between IonTorrent and re-analysis by CLC (rho.c.est = 0.9957) and similarly between GS Junior's AVA data and CLC_AVA (rho.c.est = 0.9953), verifying a strong overlap between platform-specific proprietary software and open third-party tools. There was a poor concordance between IonTorrent software and GS Junior's AVA pipeline for matched EGFR SNV frequencies, rho.c est = 0.7718.

**Table 1:** EGFR mutational analysis of lung adenocarcinoma, malignant melanoma and colorectal carcinoma – Sanger/q-PCR sequencing versus NGS.

Patient		EGFR		q-PCR	Sanger	IonTorrent			CLC_IonTorrent			454_AVA			CLC_AVA		
ID	Location	Exon	AA			SNV/DEL	Frequency	Coverage	SNV/DEL	Frequency	Coverage	SNV/DEL	Frequency	Coverage	SNV/DEL	Frequency	Coverage
3	55249063	EX20	AA787			G/A	47.4	483	G/A	49.1	568	G/A	51	2852	G/A	45.7	1959
LA																	
4	55249063	EX20	AA787			G/A	96.4	505	G/A	97	606	G/A	100	1474	G/A	100	1393
LA	55249005	EX20	AA768			G/T	5	540	G/T	5.1	529						
5	55249005	EX20	AA768			G/T	12.6	420	G/T	12.6	412						
LA	55249063	EX20	AA787			G/A	64.8	409	G/A	64.1	418	G/A	49.5	2577	G/A	50.6	2457
6	55249063	EX20	AA787			G/A	43.8	185	G/A	49.5	246	G/A	40.1	1373	G/A	49.3	2095
LA																	
7	55242464	EX19	745_750	Del	Del	Del	23.9	1404	Del	25.9	1234	Del	20.2	4197	Del	19.6	3782
LA	55249063	EX20	AA787			G/A	39.3	707	G/A	39.8	763	G/A	38.9	2298	G/A	38.7	2203
8	55241707	EX18	AA719	AA719	AA719	G/T	50.8	665	G/T	50.8	667	G/T	39.9	1969	G/T	40.6	1850
LA	55249005	EX20	AA768	AA768	AA768	G/T	26.9	750	G/T	25.4	724	G/T	43.5	550	G/T	43.4	518
	55249063	EX20	AA787			G/A	99.6	770	G/A	99.8	801	G/A	100	550	G/A	100	518
9	55241707	EX18	AA719			G/T	40.9	71	G/T	32.6	89						
LA	55249005	EX20	AA768			G/T	4.7	472	G/T	9.6	489						
	55249063	EX20	AA787			G/A	68	546	G/A	68.4	557	G/A	7	185	G/A	6.9	175
	55259450	EX21	AA836			C/T	10.9	478	C/T	10.4	450	C/T	36.7	507	C/T	36.5	480
	55259515	EX21	AA858	AA858	AA858	T/G	26.1	111	T/G	11.3	265	T/G	49.3	507	T/G	46.8	453
10	55242464	EX19	745_750	Del	Del	Del	80.3	1953	Del	82.9	3633	Del	79.3	2218	Del	79.3	2033
LA	55249063	EX20	AA787			G/A	99.5	1047	G/A	99.4	1332	G/A	100	7098	G/A	99.9	6700
	55249110	EX20	AA803			G/A	4.2	2051									
1	55259450	EX21	AA836			C/T	47.8	695	C/T	46.7	676	C/T	56.4	2984	C/T	43.5	2936
MM																	
2	55241707	EX18	AA719			G/T	27.7	170	G/T	25.7	183						
MM	55249063	EX20	AA787			G/A	99	294	G/A	99.1	319	G/A	97	1275	G/A	97.2	1230
	55249114	EX20	AA804						A/G	9.2	806						
11	55249063	EX20	AA787			G/A	4	149									
CRC																	
12	55241707	EX18	AA719			G/T	8.8	239	G/T	7.9	242						
CRC	55249063	EX20	AA787			G/A	34.4	270	G/A	32.4	318						
13 LA	55249063	EX20	AA787			G/A	44.1	68				G/A	52.5	972	G/A	52.5	947

The EGFR exons 18–21 were sequenced and compared by four methods. NGS confirmed activating mutations in *EGFR* concordant with gold standard methods, as well as other mutational variants. CLC software analysis was performed to confirm or deny base calls. Del:deletion; EX:Exon; **LA: lung adenocarcinoma; MM: malignant melanoma; CRC: colorectal cancer.**

#### Malignant melanoma (ID.1 and ID.2)

The therapeutic TKI target in EGFR exon 18 aa719 was detected in ID.2 by IonTorrent and CLC_IonTorrent analysis, albeit at the least stringent threshold of 71. No other mutations in *EGFR* were considered as they have previously been described as silent germline polymorphisms [10], [11] or did not meet the variant ‘passed’ criteria (exon 20 aa804) (Table 1).

#### Colorectal carcinoma (ID.11-ID.13)

In 1/3 clinical samples, IonTorrent and CLC_IonTorrent analysis identified a SNV at exon 18 G/T aa719 however application of the two highest stringency thresholds excluded this base call. Again, the germline silent mutation in EGFR exon 20 aa787 was returned from the analysis in all colorectal carcinoma samples in this study and has been reported by others [12] (Table 1).

### BRAF and KRAS analysis

#### Malignant melanoma (ID.1 and ID.2)

BRAF: The standard-of-care mutation, exon 15 A/T aa600 [5] was detected in both malignant melanoma clinical samples by the q-PCR method. This finding was concordant with sequence data generated from the IonTorrent NGS platform (ID.1 and ID.2) and GS Junior (ID.2 only), Table 2. Unfortunately, the clinical sample, ID.1, was exhausted and analysis with 454 GS Junior platform could not be completed.

**Table 2:** BRAF and KRAS mutational analysis of lung adenocarcinoma, malignant melanoma and colorectal carcinoma by NGS LA: lung adenocarcinoma; MM: malignant melanoma; CRC: colorectal cancer.

Patient				q-PCR	IonTorrent			CLC_IonTorrent			454_AVA			CLC_AVA		
ID	Location	Exon	AA		SNV/DEL	Frequency	Coverage	SNV/DEL	Frequency	Coverage	SNV/DEL	Frequency	Coverage	SNV/DEL	Frequency	Coverage
1	140453136	BRAF Ex15	AA600	AA600	A/T	19.4	1127	A/T	19	1098		-			-	
MM	25398284	KRAS Ex 2	AA12		C/T	5.1	9394									
2 MM	140453136	BRAF Ex15	AA600	AA600	A/T	19.5	934	A/T	19	916	A/T	9.6	14911	A/T	9.6	14278
3 LA	25398284	KRAS Ex 2	AA12	AA12	C/T	15.8	1550	C/T	15.7	1551	C/T	13.6	7544	C/T	13.5	6209
4 LA																
5 LA																
6 LA	25398285	KRAS Ex 2	AA12	AA12	C/A	49.5	812	C/A	49.6	790	C/A	60.9	20845	C/A	39.5	17036
7 LA																
8	25398234	KRAS Ex 2	AA6								C/T	6.4	7047	C/T	6.2	6549
LA	25398280	KRAS Ex 2	AA14								G/A	5.6	7044	G/A	5.7	6489
	25398303	KRAS Ex 2	AA29								G/A	6.8	7045	G/A	5.7	6027
9 LA	25398279	KRAS Ex 2	AA14								C/T	6.6	10054	C/T	6.2	9253
10 LA																
11 CRC																
12 CRC	140453136	BRAF Ex15	AA600		A/T	4.38	1552									
13 CRC																

KRAS: Although not meeting the variant ‘passed’ criteria, it is noteworthy that the clinically relevant *KRAS* mutation in exon 2 C/T aa12 was called (IonTorrent only) in ID.1, though at a low frequency of 5.1%. This variant was not detected by q-PCR sequencing of *KRAS*, Table 2.

#### Lung adenocarcinoma (ID.3-ID.10)

BRAF: No mutations in the *BRAF* gene were detected, concordant across all technologies.

KRAS: The important therapeutic *KRAS* mutation in colon cancer was detected in ID.3 and ID.6 (exon 2 aa12), two EGFR wildtype samples. Furthermore, the detection of *KRAS* mutations by NGS was concordant with the gold standard methods, Table 2. Within exon 2 of the *KRAS* gene, the GS Junior AVA software (and CLC_AVA) but not IonTorrent, called other variants with frequencies ≤7%, including a SNV at exon 2 aa14 with a COSMIC ID (http://www.sanger.ac.uk/perl/genetics/CGP/cosmic?action=bygene&ln=KRAS&start=4&end=20&coords=AA%3AAA) (Table 2). Interestingly, these multiple variants were only present in ID.8 and ID.9 both of which harbour several *EGFR* activating mutations (Table 1).

#### Colorectal carcinoma (ID.11-ID.13)

All colorectal carcinoma samples had been reported as *BRAF* and *KRAS* wildtype by q-PCR sequencing. For KRAS analysis, this was concordant across all technologies, however the *BRAF* mutation at codon 600 was called in 1/3 colorectal carcinoma samples though at a low coverage equal to 4.38% and only by 1/4 analysis (IonTorrent, Table 2).

### Full analysis of IonTorrent AmpliSeq panel

#### Lung adenocarcinoma (ID.3-ID.10)

**Figure 1:**
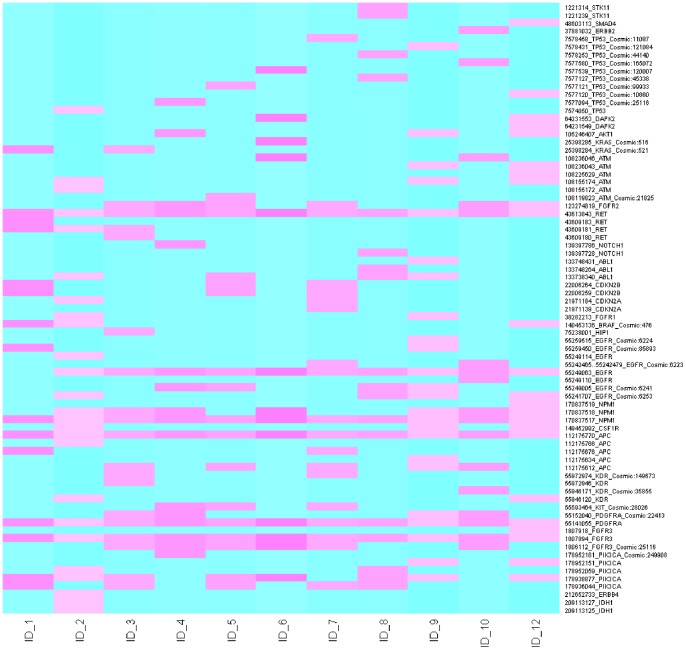
Distribution of variants detected using the Ion AmpliSeq Cancer Panel. A heat map was generated illustrating the variants occurring in all 46 genes by both IonTorrent software versions in each of the clinical samples. COSMIC tracked variants are also described.

As defined before, variants ‘passed’ if present in 2/4 analyses: IonTorrent V2.0.1, CLC_V2.0.1, IonTorrent V2.2 and CLC_V2.2, Figure 1. Several SNVs and at different genomic positions, demonstrated by the heat map ‘distribution of variants’, were observed in TP53 in 87.5% of lung adenocarcinomas (Figure 1, Table 3). Even at the highest stringency threshold (QBVDiii = 500; Table 3), TP53 SNVs were called in 62.5% of samples. This is improbable, as when we compare this frequency with the COSMIC database for TP53 mutations in lung adenocarcinoma, the results are discordant, Table 3. Five genes were flagged as mutant in all 8 lung adenocarcinoma samples namely RET, APC, FGFR3, NPM1 and PDGFRA when the least stringent QBVD threshold was applied (QBVDi = 71). By applying the highest threshold (QBVDiii = 500), still 100% of samples had mutations in PDGFRA and APC, while 37.5% and 75% of patient samples had FGFR3 and RET mutations, respectively. For each of the five genes, the findings are markedly discordant with COSMIC frequencies (Table 3) and were regarded as false-positives. Additionally, SNVs in NPM1 were disregarded as detection was within a homopolymer region, a documented caveat of the IonTorrent variant calling software [13]. Here, the NGS methodology needs further software and chemistry improvements. The importance of applying thresholds was addressed in relation to other genes on the panel. A low stringency level (QBVDi = 71) allowed the detection of STK11, DAPK2, CDKN2A, CDKN2B, HIP1 and CSF1R SNVs in one or two (CDKN2B only) of the patient samples. Again, these variants have not been considered in the final gene profile for lung adenocarcinomas as they are discordant with that reported by the COSMIC database (Figure 1, Table 3). Other SNVs that were detected, even by a highly stringent threshold approach, but excluded when referenced against the COSMIC database included PIK3CA (62.5% of samples vs 2.2% COSMIC frequency), KIT (37.5% vs 0.3%), KDR (37.5% vs 4%), ABL1 (37.5% vs 0.8%), NOTCH (25% vs 1.5%), FGFR2 (62.5% vs 1.1%) and ATM (50% vs 5%). Mutations that could be genuine but would require further investigation in a larger patient cohort are AKT1, FGFR1 and ERBB2, each occurring in 1/8 of the clinical samples and are reported as low frequency occurring mutations in lung adenocarcinoma by the COSMIC database (Figure 1, Table 3).

**Table 3:** NGS gene mutation detection using the Ion AmpliSeq Cancer Panel in lung adenocarcinoma.

Gene	QBVDi = 500	QBVDii = 259	QBVDiii = 71	Cosmic %
PIK3CA	62.5	75	87.5	0.022
FGFR3	37.5	75	100	0
PDGFRA	100	100	100	4
KIT	37.5	37.5	37.5	0.003
KDR	37.5	37.5	37.5	4
APC	100	100	100	0.033
CSF1R	0	0	12.5	0.012
NPM1	50	75	100	0
HIP1	0	0	12.5	0
FGFR1	0	12.5	12.5	0.007
CDKN2A	0	0	12.5	0.122
CDKN2B	0	0	25	0.005
ABL1	37.5	37.5	37.5	0.008
NOTCH1	25	25	25	0.015
RET	75	100	100	0.014
FGFR2	62.5	62.5	62.5	0.011
ATM	50	50	50	5
AKT1	0	12.5	25	0.002
DAPK2	0	0	12.5	0
TP53	62.5	75	87.5	36
ERBB2	12.5	12.5	12.5	3
STK11	0	0	12.5	10

The proportion (%) of lung adenocarcinoma samples harbouring mutations in the other genes interrogated by the panel and the resultant application of different detection thresholds. Frequencies were compared with that observed in the COSMIC database.

#### Colorectal carcinoma (ID.11-ID.13)

Analysis of ID.12 was carried out using IonTorrent V2.01 and V2.2 as this sample was sequenced prior to the software upgrade, hence included in the heat map generated in Figure 1. ID.11 and ID.13 have been analysed by V2.2 only, Table 4. Due to the limited numbers available, we adjusted our inclusion criteria for the additional genes interrogated by the Ion AmpliSeq panel. In each of the colorectal carcinoma samples, SNVs were considered if a) detected in 2/3 samples tested and b) called by both IonTorrent analysis and CLC re-analysis. With this approach, FGFR3, PDGFRA, APC, RET, ATM and TP53 were flagged; however, experience in the larger lung adenocarcinoma cohort (Table 3) may call into question the reliability of the former 4 genes, Table 4. The results of ATM and TP53 do not allow analytical comment within this small sample number.

**Table 4:** NGS gene mutation detection using the Ion AmpliSeq Cancer Panel in colorectal carcinoma.

ID_11				ID_13			
Ion Torrent 2.2		CLC_Ion Torrent		Ion Torrent 2.2		CLC_Ion Torrent	
Location	Gene	Location	Gene	Location	Gene	Location	Gene
		209113123	IDH1			178927969	PIK3CA
		209113125	IDH1			178927972	PIK3CA
		178927969	PIK3CA			178938877	PIK3CA
		178927970	PIK3CA	1807894	**FGFR3**	1807894	**FGFR3**
		178927972	PIK3CA			1808323	**FGFR3**
1807894	**FGFR3**	1807894	**FGFR3**	55141055	**PDGFRA**	55141055	**PDGFRA**
		1807904	**FGFR3**	55593481	KIT	55593481	KIT
55141055	**PDGFRA**	55141055	**PDGFRA**	153247311	FBXW7 (DEL)		
		55152040	**PDGFRA**			153247316	FBXW7
55972974	KDR	55972974	KDR	112175770	**APC**	112175770	**APC**
		153258992	FBXW7				
		112175193	**APC**	55249063	EGFR		
112175770	**APC**	112175770	**APC**			116339643	MET
112175952	**APC**					38282213	FGFR1
55249063	EGFR			43613843	**RET**	43613843	**RET**
		38282202	FGFR1			43609181	**RET**
		38282213	FGFR1			89717599	PTEN
43613843	**RET**	43613843	**RET**	123274818	FGFR2		
		123274819	FGFR2	123274819	FGFR2		
		108155172	**ATM**	108123531	**ATM**		
		108155174	**ATM**			108155172	**ATM**
		108218107	**ATM**			108155174	**ATM**
		108236190	**ATM**	108173659	**ATM**		
		108236194	**ATM**			108218107	**ATM**
1207084	STK11			48923143	RB1 (DEL)		
				7578263	**TP53**	7578263	**TP53**
				48604689	SMAD4 (DEL)		
				1207065	STK11		
				1207084	STK11		

In conjunction with Figure 1 (ID.12), the genes in bold text occurred in 2/3 samples. ID.12 was sequenced prior to the software upgrade (V2.01, V2.2). ID.11 and ID.13 by V2.2 only.

#### Malignant melanoma (ID.1 and ID.2)

As above, we adjusted the inclusion criteria. SNVs were considered if detected in both malignant melanoma (*BRAF* mutant) samples. Genes included FGFR3, PDGFRA, APC, RET, NPM1 and PIK3CA. As before, the reliability of the former 4 genes is questionable. NPM1 was disregarded as the mutation was flagged in a homopolymer region. PIK3CA is a likely true mutation identified by the Ion AmpliSeq panel (Figure 1) and requires future validation.

### Threshold filtering

The gene information obtained from interrogation of the Ion AmpliSeq panel was represented as a box plot (Figure S1) demonstrating the importance of threshold application in SNV detection in NGS. The lines represent each of the QBVD thresholds (i = 71, ii = 259, iii = 500) and the proportion of gene SNVs that are filtered according to what stringency level has been applied.

**Figure 2:**
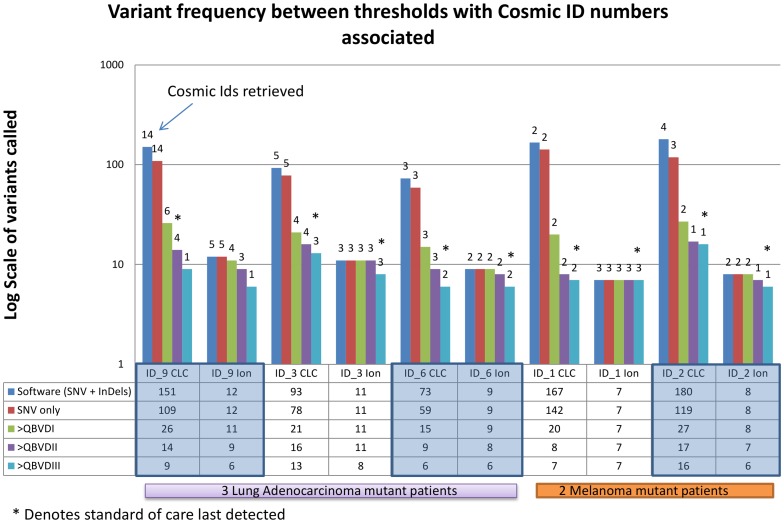
Variant frequency between thresholds with Cosmic IDs associated. The graph illustrates the importance of filtering mutations by applying different detection thresholds in the bioinformatics analysis. ID.1, 2, 3, 6 and 9 were selected as they contained important standard-of-care SNVs.

Figure 2 demonstrates the relevance of ‘filtering by threshold application’ of COSMIC SNVs in some of those patients with standard-of-care mutations in *EGFR* (ID.9), *KRAS* (ID.3 and ID.6) and *BRAF* (ID.1 and ID.2). For example, in ID.9 the lowest threshold level of detection for large deletions, calls variants in 151 gene regions in the Ion AmpliSeq cancer panel, 14 of which have been referenced in the COSMIC database. By applying an internal SNV only detection capability in CLC, the number of SNVs called in the full panel was reduced to 109 (14 COSMIC references still remained). As expected, application of the QBVD thresholds (i = 71, ii = 259 and iii = 500) resulted in a decrease in the number of SNVs detected from 26 to 14 to 9 and those that were COSMIC tracked, reduced from 6 to 4 to 1, respectively. An interesting observation of this threshold approach is that clinically important mutations in *KRAS* (ID.3 and ID.6) and *BRAF* (ID.1 and ID.2) were still detected when a QBVDiii (500) was applied, however, the clinically relevant mutation in *EGFR* would not have been reported by applying this threshold, Figure 2.

## Discussion

Generation of numerous DNA reads from significant portions of the genome in little time will transform the way we interrogate DNA in cancer diagnostics. The sooner NGS is fully fit for this purpose, the easier it will be to interrogate numerous possible drug targets per patient in a time-sensitive manner, and thus, design broader short-term and long-term therapeutic strategies.

In our opinion, the current study has 4 main points of interest. Firstly, NGS is reliable in detecting known standard-of-care mutations with good sensitivity and specificity within our small sample panel. For example, deletions in *EGFR* exon 19 and SNVs in *EGFR* exon 18 aa719, exon 20 aa768 and exon 21 aa858 in lung adenocarcinoma [8]; *KRAS* SNVs in exon 2 aa12 in 50% of wildtype *EGFR* lung adenocarcinoma [14] and *BRAF* mutations at exon 15 aa600 in DNA from malignant melanoma [15] were all accurately identified by NGS, concordant with conventional mutation detection methods.

Secondly, NGS called other mutations in *EGFR, KRAS* and *BRAF* that represent standard-of-care but were undetected by Sanger/q-PCR methods. This may be due to a) an increased sensitivity of NGS or b) a lack of specificity of NGS. For example, our preliminary dilution sensitivity tests for NGS, prior to the validation of the technology, allowed us to indicate that the standard-of-care mutation in *EGFR* exon 21 aa858 was detected at 1% in a mix of wildtype/mutant DNA from a cell line (data not shown); however other mutations were also detectable at this level, suggesting that the sensitivity assays are unlikely to reflect DNA extracted from FFPE, thus making the direct correlation of NGS sensitivity with that calculated for Sanger and q-PCR approaches, 10% and 5% respectively, questionable. In any case, it is likely that many of these new mutations are not genuine and thus further refinement of the technology is necessary.

Thirdly, the need for a better NGS technology is also a consequence of the results obtained with the other 43 genes. Again, it was out of the scope of this work to Sanger sequence every mutation identified in the NGS analysis, and this is indeed one of the limitations of our study. However, the approximation to COSMIC tells us that for many of them, the current technology may be over-calling mutations. This, which may be acceptable for discovery studies where significant downstream validations need to take place, is not appropriate in the context of routine cancer diagnostics.

Fourthly, our study is a clear example of how the application of new technologies to patient care will be dictated by bioinformatics approaches as much as wet-bench related work. The importance of the bioinformatics threshold approach in identifying credible results is a clear illustration of this and calls for the presence of *molecular diagnostic bioinformaticians* embedded in future reference molecular diagnostic operations.

No doubt as NGS technologies (and bioinformatics tools) evolve, accuracy will be enhanced thereby meeting our two provisos: a) NGS technologies are as efficient as the current detection methods in the diagnosis of those single genes that, for a given cancer type, represent standard-of-care; and b) the extra information that is generated in the process is of sufficient quality to consider alternative therapies or be accepted for future research endeavours. In future validations of NGS technology, one must deal with the added benefits of the discovery of new mutations versus the potential false positives that can result from altering the threshold. The importance of applying thresholds has been investigated here. In the situation where we observe lower frequency (than the QBVDiii = 500), it is likely that the mutation occurs in a small population of tumour cells or that the actual sample contained many stromal cells for example, thereby diluting the mutation frequency. The benefits of NGS are that the technology is sensitive enough to detect mutations at low frequency and in mixed tumour DNA samples; in such cases the threshold must be lowered to detect this. We believe that the sequencing of tumour samples for diagnostics must be carried on in parallel with the sequencing of an adjacent histologically normal sample; the latter acting as a baseline reference that should eliminate false positives, reveal germline mutations in both samples and finally reveal the true mutational profile of that tumour sample. Investment into sequencing precision, accuracy, reliability and bioinformatics will accelerate NGS integration into clinical cancer diagnostics either as a parallel tool with conventional sequencing methods or, in time, as a stand-alone approach to mutation detection.

## Supporting Information

Figure S1 **The boxplot represents the distribution of mutation variants, by coverage, obtained using the Ion AmpliSeq Cancer Panel and analyzed by IonTorrent V2.2 and CLC_V2.2.** The lines represent QBVD thresholds (i, ii, iii) demonstrating the number of variants filtered depending on the level of detection applied.
(TIFF)

Table S1 **DNA selected for NGS analysis.**
(DOCX)

File S1 **Ethics statement and DNA Sample Collection.**
(DOC)

## References

[1] Sanger F, Nicklen S, Coulson AR (1977) DNA sequencing with chain-terminating inhibitors. Proc Natl Acad Sci U S A 74: 5463–5467. 271968 10.1073/pnas.74.12.5463 PMC431765

[2] Martinez DA, Nelson MA (2010) The next generation becomes the now generation. PLoS Genet 8 6(4): e1000906. 10.1371/journal.pgen.1000906 PMC2851573 20386747

[3] Meldrum C, Doyle MA, Tothill RW (2011) Next-generation sequencing for cancer diagnostics: A practical perspective. Clin Biochem Rev 32: 177–195. 22147957 PMC3219767

[4] Choi M, Scholl UI, Ji W, Liu T, Tikhonova IR, et al (2009) Genetic diagnosis by whole exome capture and massively parallel DNA sequencing. Proc Natl Acad Sci U S A 106: 19096–19101 10.1073/pnas.0910672106. 19861545 10.1073/pnas.0910672106 PMC2768590

[5] Chapman PB, Hauschild A, Robert C, Haanen JB, Ascierto P, et al (2011) Improved survival with vemurafenib in melanoma with BRAF V600E mutation. N Engl J Med 364: 2507–2516 10.1056/NEJMoa1103782. 21639808 10.1056/NEJMoa1103782 PMC3549296

[6] Ascierto PA, Kirkwood JM, Grob JJ, Simeone E, Grimaldi AM, et al (2012) The role of BRAF V600 mutation in melanoma. J Transl Med 10: 85 10.1186/1479-5876-10-85. 22554099 10.1186/1479-5876-10-85 PMC3391993

[7] Lynch TJ, Bell DW, Sordella R, Gurubhagavatula S, Okimoto RA, et al (2004) Activating mutations in the epidermal growth factor receptor underlying responsiveness of non-small-cell lung cancer to gefitinib. N Engl J Med 350: 2129–2139 10.1056/NEJMoa040938. 15118073 10.1056/NEJMoa040938

[8] Sharma SV, Bell DW, Settleman J, Haber DA (2007) Epidermal growth factor receptor mutations in lung cancer. Nat Rev Cancer 7: 169–181 10.1038/nrc2088. 17318210 10.1038/nrc2088

[9] Lievre A, Bachet JB, Le Corre D, Boige V, Landi B, et al (2006) KRAS mutation status is predictive of response to cetuximab therapy in colorectal cancer. Cancer Res 66: 3992–3995 10.1158/0008-5472.CAN-06-0191. 16618717 10.1158/0008-5472.CAN-06-0191

[10] Sikkema-Raddatz B, Johansson LF, de Boer EN, Almomani R, Boven LG, et al. Targeted Next-Generation Sequencing can Replace Sanger Sequencing in Clinical Diagnostics. Hum Mutat. 2013 Apr 8. doi: 10.1002/humu.22332. [Epub ahead of print]. 10.1002/humu.22332 23568810

[11] Conde E, Angulo B, Tang M, Morente M, Torres-Lanzas J, et al (2006) Molecular context of the EGFR mutations: Evidence for the activation of mTOR/S6K signaling. Clin Cancer Res 12: 710–717 10.1158/1078-0432.CCR-05-1362. 16467080 10.1158/1078-0432.CCR-05-1362

[12] Schmid K, Oehl N, Wrba F, Pirker R, Pirker C, et al (2009) EGFR/KRAS/BRAF mutations in primary lung adenocarcinomas and corresponding locoregional lymph node metastases. Clin Cancer Res 15: 4554–4560 10.1158/1078-0432.CCR-09-0089. 19584155 10.1158/1078-0432.CCR-09-0089

[13] Metzger B, Chambeau L, Begon DY, Faber C, Kayser J, et al (2011) The human epidermal growth factor receptor (EGFR) gene in european patients with advanced colorectal cancer harbors infrequent mutations in its tyrosine kinase domain. BMC Med Genet 12: 144 10.1186/1471-2350-12-144. 22026926 10.1186/1471-2350-12-144 PMC3215960

[14] Loman NJ, Misra RV, Dallman TJ, Constantinidou C, Gharbia SE, et al (2012) Performance comparison of benchtop high-throughput sequencing platforms. Nat Biotechnol 30: 434–439 10.1038/nbt.2198. 22522955 10.1038/nbt.2198

[15] Pao W, Wang TY, Riely GJ, Miller VA, Pan Q, et al (2005) KRAS mutations and primary resistance of lung adenocarcinomas to gefitinib or erlotinib. PLoS Med 2: e17 10.1371/journal.pmed.0020017. 15696205 10.1371/journal.pmed.0020017 PMC545207

