# Supplementary figures and images for: Validation of Next Generation Sequencing Technologies in Comparison to Current Diagnostic Gold Standards for BRAF, EGFR and KRAS Mutational Analysis
Source: PLoS One. 2013 Jul 26;8(7):e69604. doi: 10.1371/journal.pone.0069604 (PMC3724913; doi:10.1371/journal.pone.0069604)

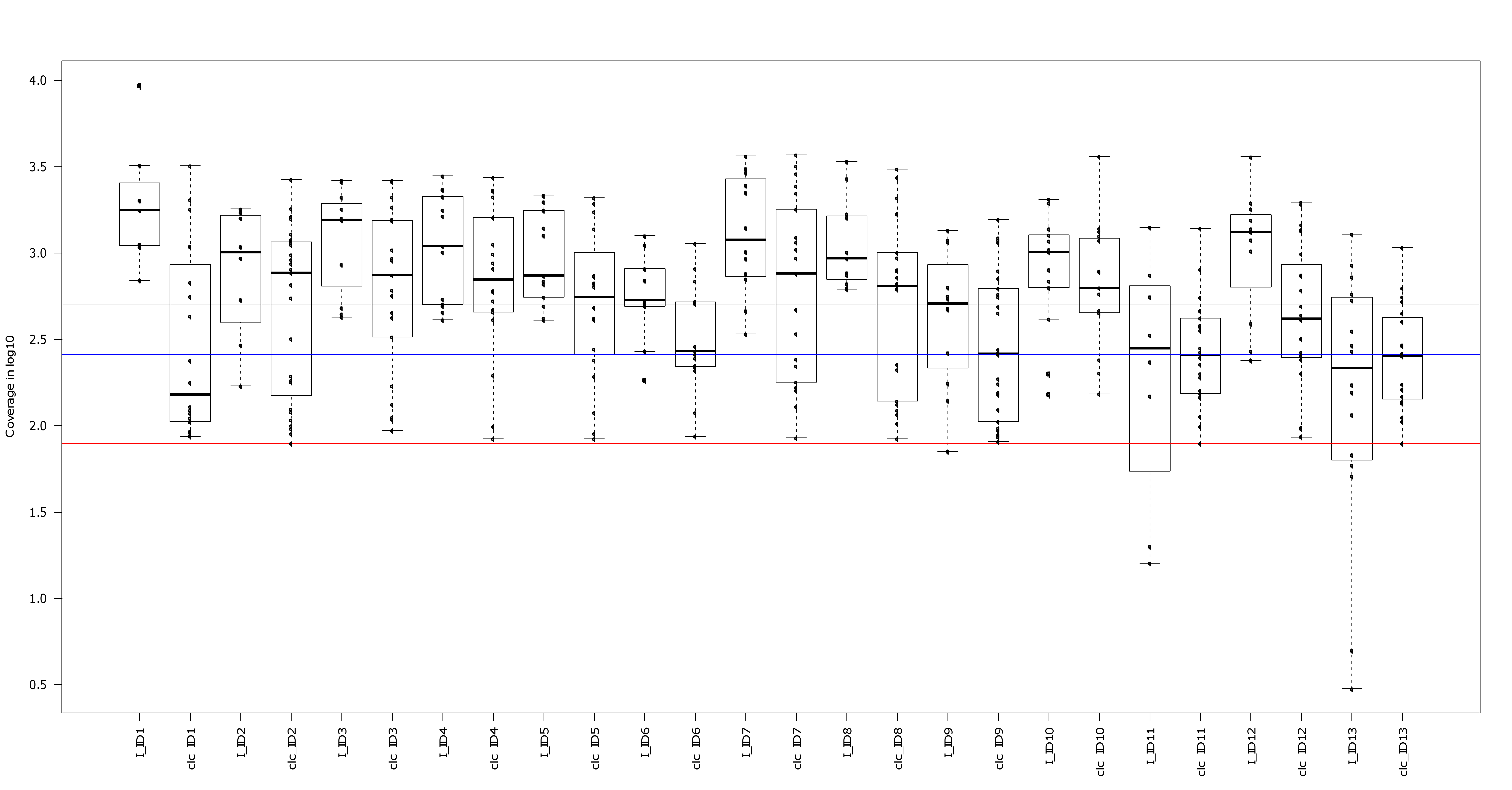

Supplement: Figure S1 — The boxplot represents the distribution of mutation variants, by coverage, obtained using the Ion AmpliSeq Cancer Panel and analyzed by IonTorrent V2.2 and CLC_V2.2. The lines represent QBVD thresholds (i, ii, iii) demonstrating the number of variants filtered depending on the level of detection applied. (TIFF) [file pone.0069604.s001.tiff]
