# Supplementary material for: Validation of Next Generation Sequencing Technologies in Comparison to Current Diagnostic Gold Standards for BRAF, EGFR and KRAS Mutational Analysis
Source: PLoS One. 2013 Jul 26;8(7):e69604. doi: 10.1371/journal.pone.0069604 (PMC3724913; doi:10.1371/journal.pone.0069604)
Supplement: Table S1 — DNA selected for NGS analysis. (DOCX) [file pone.0069604.s002.docx]

| Patient No. | Cancer Type | BRAF status | EGFR status | KRAS status | Qubit® DNA concentration (ng/μl) |
| --- | --- | --- | --- | --- | --- |
| 1 | Melanoma | Mutant |  |  | 10.9 |
| 2 | Melanoma | Mutant |  |  | 22.2 |
| 3 | Lung |  | Wildtype |  | 31.7 |
| 4 | Lung |  | Wildtype |  | 3.4 |
| 5 | Lung |  | Wildtype |  | 20.6 |
| 6 | Lung |  | Wildtype |  | 45.9 |
| 7 | Lung |  | Mutant |  | 25.4 |
| 8 | Lung |  | Mutant |  | 10.0 |
| 9 | Lung |  | Mutant |  | 26.4 |
| 10 | Lung |  | Mutant |  | 14.6 |
| 11 | Colon |  |  | Wildtype | 37.3 |
| 12 | Colon |  |  | Wildtype | 28.7 |
| 13 | Colon |  |  | Wildtype | 26.7 |

**Table S1. DNA selected for NGS analysis**
