# Supplementary material for: Validation of Next Generation Sequencing Technologies in Comparison to Current Diagnostic Gold Standards for BRAF, EGFR and KRAS Mutational Analysis
Source: PLoS One. 2013 Jul 26;8(7):e69604. doi: 10.1371/journal.pone.0069604 (PMC3724913; doi:10.1371/journal.pone.0069604)
Supplement: File S1 — Ethics statement and DNA Sample Collection. (DOC) [file pone.0069604.s003.doc]

Ethics statement and DNA sample collection

Thirteen aliquots of tumour DNA extracted from formalin-fixed-paraffin-embedded (FFPE) malignant melanoma, lung adenocarcinoma and colon carcinoma, and genotyped by Sanger/q-PCR sequencing for BRAF, EGFR and KRAS status, respectively, were obtained from the Northern Ireland Biobank following ethics approval (NIB12-0049) (See Table S1). Prior to sequencing, extraction was performed using a Qiagen DNA extraction kit (Qiagen) according to the manufacturer’s instructions. DNA concentrations were quantified using a Qubit®dsDNA HS Kit on the Qubit®2.0 fluorometer (Invitrogen, Life Technologies), outlined in the manufacturer’s instructions. DNA purity had been assessed by absorbance ratios at A260/A280 on the Nanodrop (Thermo Scientific) (data not shown).

Sanger Sequencing-BRAF/ KRAS/EGFR

BRAF (exon 15), KRAS (exon 2) and EGFR (exon 18-21) PCR products were purified with ExoSap-IT (Affymetrix). Amplicon quality was assessed by electrophoresis on a 2% agarose gel (data not shown) and quantity determined using a Nanodrop. Purified PCR products were sequenced using the BigDye Terminator version 3.1 Cycle Sequencing Kit, according to the manufacturer’s instructions (Applied Biosystems, Life Technologies). The 3500 Genetic Analyzer (Applied Biosystems) was used for capillary electrophoresis and sequence analysis. All samples were sequenced in both directions.

qPCR-based sequencing-BRAF/ KRAS/EGFR

A real-time PCR mutation screen was carried out on each sample using the Cobas®4800 BRAF V600, Cobas®KRAS Mutation Test and Cobas®EGFR Mutation Test, according to the manufacturer’s instructions (Roche).

Sequencing workflow

Preparation of DNA for NGS amplicon sequencing was similar for both the Ion Torrent PGM (Life Technologies) and the 454 GS Junior (Roche) instruments. In brief, experimental stages consisted of DNA amplicon library preparation, emulsion PCR (emPCR), enrichment and sequencing. Sequence data was mapped and aligned against the HG19 reference and variants were called using platform-specific proprietary software analysis and third-party independent bioinformatics software tools. By way of comparison across all sequencing platforms, each DNA sample was sequenced by Sanger and/or q-PCR (Cobas, Roche), Ion Torrent PGM and Roche 454 GS Junior to interrogate each of the standard-of-care models *BRAF, EGFR* and *KRAS,* in every case. In addition, a further 43 genes were analysed with the Ion Torrent PGM as part of a ready-made kit (Ion AmpliSeq™ Cancer Panel).
